# Circulating Irisin Levels Are Not Affected by Coffee Intake: A Randomized Controlled Trial

**DOI:** 10.1371/journal.pone.0094463

**Published:** 2014-04-11

**Authors:** Patricia R. Peter, Kyung Hee Park, Joo Young Huh, Nicole M. Wedick, Christos S. Mantzoros

**Affiliations:** 1 Department of Internal Medicine, Beth Israel Deaconess Medical Center, Harvard Medical School, Boston, Massachusetts, United States of America; 2 Division of Endocrinology, Diabetes, and Metabolism, Beth Israel Deaconess Medical Center, Harvard Medical School, Boston, Massachusetts, United States of America; 3 Department of Family Medicine, Hallym University Sacred Heart Hospital, Hallym University, Gyeonggi-do, Korea; 4 Division of Preventive and Behavioral Medicine, Department of Medicine, University of Massachusetts Medical School, Worcester, Massachusetts, United States of America; 5 Section of Endocrinology, Boston VA Healthcare System, Harvard Medical School, Boston, Massachusetts, United States of America; Indiana University Richard M. Fairbanks School of Public Health, United States of America

## Abstract

Irisin, secreted by skeletal muscle and possibly fat, is hypothesized to play an important role in modulating energy expenditure, obesity and metabolism. Coffee consumption also increases energy expenditure and leads to positive metabolic effects, but whether these effects are mediated by irisin remains unknown. The objective of this study was to determine the association between baseline irisin levels and the metabolic profile in humans and to investigate whether consumption of caffeinated coffee alters irisin levels. To this end, a secondary analysis was performed investigating irisin levels at baseline and after eight weeks in 32 healthy, overweight coffee drinkers who were randomized to consumption of 5 cups per day of instant caffeinated coffee, decaffeinated coffee, or water. Spearman correlation and analysis of covariance analyses were performed to identify possible associations. Irisin levels were positively correlated with waist circumference (r = 0.41, p = 0.02), fat mass (r = 0.44, p = 0.01) and CRP (r = 0.47, p = 0.007). Though there was a trend towards increased levels of irisin over time in the caffeinated coffee group (+1.8%) when compared to the placebo group (−4%) this did not reach statistical significance (p = 0.75 for the trend). This first randomized trial failed to reveal any effects of coffee consumption on irisin levels, but a larger trial, appropriately sized on the basis of data provided by this study, is needed to conclusively investigate such a relationship.

**Trial Registration:**

Clinicaltrials.gov NCT00305097

## Introduction

Irisin is a novel myokine thought to play an important role in energy expenditure by mediating the exercise-induced browning of fat [1]–[3]. As the browning of adipose tissue is hypothesized to improve insulin sensitivity and decrease weight gain, irisin has become an attractive target for potential anti-obesity therapy [4]. Though higher irisin levels are associated with lower body weight and improved glucose tolerance in mice, the role of irisin in human metabolism remains unclear [1], [5]. Thus far, three cross-sectional studies in humans have demonstrated that irisin levels are lower in patients with type II diabetes, suggesting irisin could play a protective role in glucose homeostasis [4], [6], [7]. The association between irisin and body mass index (BMI) is more controversial with two studies showing a negative relationship [4], [6] and three others showing a positive association [7]–[9]. While some of this discrepancy could be attributed to the different and better validated assay used in the latter three studies, it is also possible that irisin regulation is more complex than first suspected [10]. Having reported the latter relationship between BMI and irisin in our prior study [8], we proposed that irisin could be secreted in an effort to counteract insulin resistance in the obese but that once metabolic disease occurs, irisin resistance develops, similar to several known hormone resistance syndromes [11]. A recent paper by Polyzos et al supports this hypothesis, noting that irisin appears to exhibit a positive relationship with BMI in healthy subjects but an inverse relationship in diseased states [12]. Irisin’s relationships to other important hormones including leptin and adiponectin have been inconsistent across studies as well but are potentially mediated by an underlying association between irisin and obesity [8], [9]. Ultimately, associations between irisin and metabolic factors need to be further clarified, not only via simple association studies but also using models that adjust for potential confounders such as obesity.

Coffee consumption increases energy expenditure and is thought to decrease the incidence of diabetes [13]–[16]. One proposed mechanism to explain this association is that coffee may facilitate glucose uptake by skeletal muscle via increased translocation of the GLUT4 transporter to the plasma membrane [17]–[19]. Also, coffee consumption has been associated with increased levels of adiponectin, a hormone thought to have insulin-sensitizing properties [20]–[22]. Thus, there is some evidence that caffeine increases energy expenditure and positively affects metabolism potentially via interaction with the skeletal muscle and whether irisin is a potential mediator in this process is as yet unknown.

The purpose of this study is twofold: to identify associations between irisin and markers of metabolism in humans and to determine whether coffee consumption affects irisin levels. As caffeine increases energy expenditure and irisin levels appear to rise with increased energy expenditure, we hypothesize that irisin levels will increase with coffee consumption. To this end, we have performed a secondary analysis of the serum levels of irisin in overweight coffee drinkers who were randomly assigned to consumption of caffeinated coffee, decaffeinated coffee, or water for eight weeks. This study aims to shed further light on the possible mechanisms by which irisin and coffee consumption can lead to improved health outcomes.

## Materials and Methods

### Subjects

**Figure 1:**
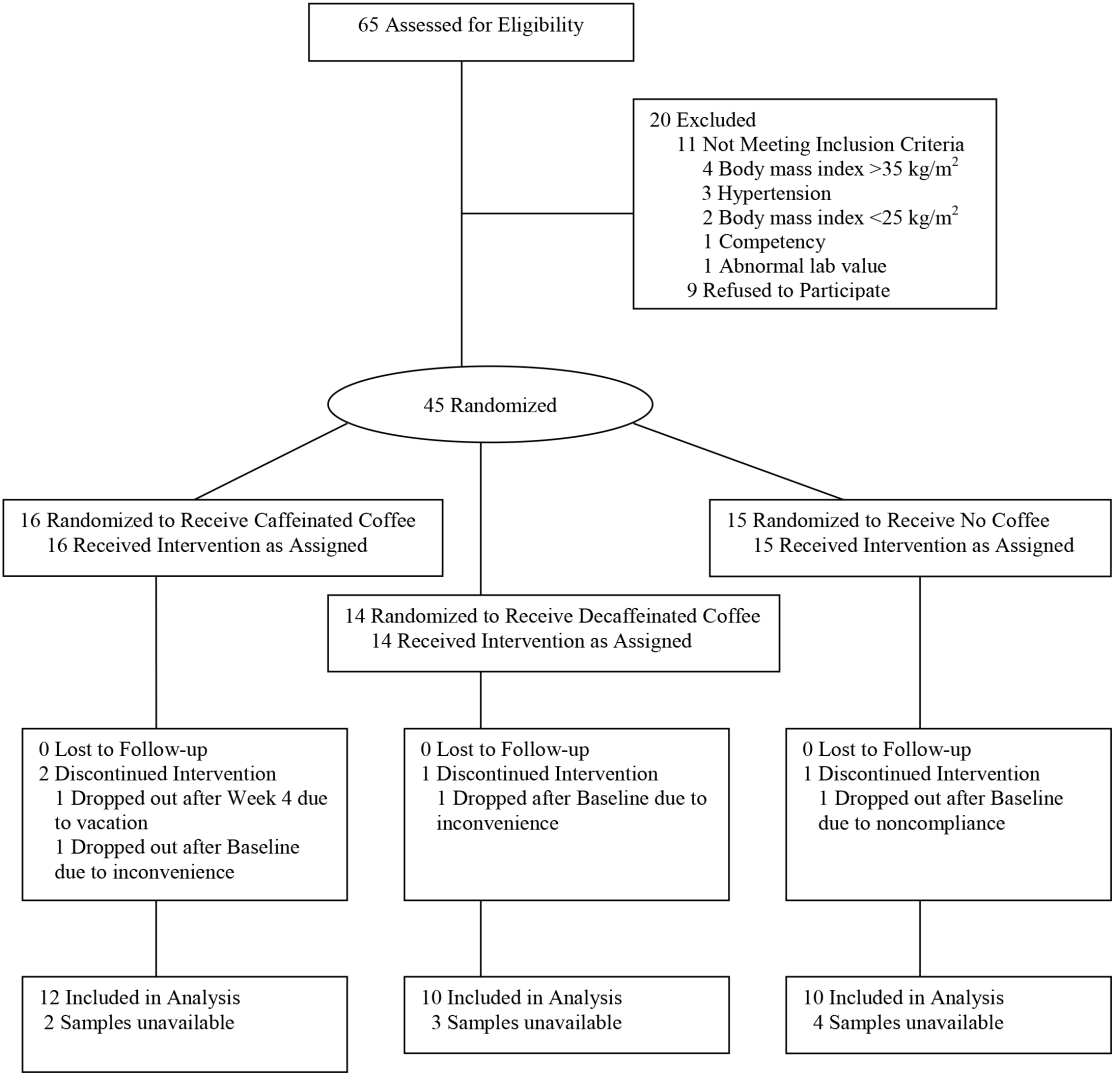
Flow of participants through the study.

Forty-one overweight (BMI 25–35 kg/m^2^) but otherwise healthy adults who were regular coffee drinkers (≥2 cups/day) were recruited between 2006 and 2008 with inclusion and exclusion criteria that have previously been described [20]. Participants were randomized via the PROC PLAN procedure in the Statistical Analysis System 9.1 (SAS Institute Inc., NC, US) to three different groups (caffeinated coffee, decaffeinated coffee, no coffee) in block sizes of six. Thirty-two subjects were analyzed in this study as the remaining samples were unavailable for assay (Figure 1). The protocol for this trial and supporting CONSORT checklist are available as supporting information (see Checklist S1 and Protocol S1).

### Ethics Statement

This study was approved by the Beth Israel Deaconess Medical Center (BIDMC) Committee on Clinical Investigations and Institutional Review Board in accordance with the Helsinki Declaration of 1975 as revised in 1983. All participants gave written informed consent prior to participation in the study. The clinical trial registration number is NCT00305097 at clinicaltrials.gov.

### Protocol

After a two-week long caffeine washout and an overnight fast, participants underwent baseline physical examination, blood draw and oral glucose tolerance test (OGTT). Participants were instructed to maintain their usual exercise and dietary habits and to abstain from caffeine-containing foods throughout the study. Participants returned after eight weeks for adherence questionnaires and repeat blood draws.

### Intervention

Each day, participants in the two coffee arms of the study consumed five two-gram portions of instant coffee (caffeinated or decaffeinated Nestlé’s Taster’s Choice) prepared with 6 ounces of boiling water. Participants randomized to the no coffee arm drank instead five 6-ounce glasses of water. As was previously reported, the five daily cups of caffeinated coffee provided 345 mg caffeine and 302 mg chlorogenic acid while five cups of decaffeinated coffee provided 216 mg chlorogenic acid [20]. At six weeks, serum caffeine levels were measured in all participants during a non-fasting blood draw to assess for compliance [20].

### Measurements

At each study visit, weight, height, and waist circumference were measured by a trained investigator according to standard definitions. Body composition was measured using Tanita (model Quantum II, Lean Body software, RJL Systems,Clinton Township, MI, US) single frequency bioelectrical impedance analysis [23], [24]. Glucose was measured by glucose hexokinase method using an autoanalyzer in the central clinical laboratory at the Beth Israel Deaconess Medical Center. The homeostasis model assessment for insulin resistance (HOMA-IR) was calculated as [(fasting glucose × fasting insulin)/405] from the OGTT [20]. All samples were stored in a liquid nitrogen freezer at <−130°C until assayed. Samples were assayed in duplicate with all samples for the same participant included in the same batch. The investigator performing the assays was blinded to the intervention group that the samples belonged in. Double-antibody radioimmunoassay (Immulite Chemiluminescence, Siemens Co., New York, NY, US) was used to measure insulin [intra-assay coefficient of variation (CV) 2.1%], C-reactive protein (CRP) (CV 3.6%), and IL-6 (CV 4.7%). Adiponectin (CV 3.9%) and irisin (CV 6.3%) levels were measured by enzyme linked immunosorbent assay kits (Millipore Corporation, Billerica, MA, US, for adiponectin; Phoenix Pharmaceuticals, Inc, Burlingame, CA, US, Cat. No. EK-067-53 for irisin) as previously described [8].

### Statistical Analysis

Comparisons of the baseline characteristics for each group were performed using χ^2^ test for categorical variables and analysis of variance for the continuous variables with normal distribution (see Figures S1–S5 to see scatter plots of the variables analyzed). Normality test was done by Shapiro-Wilk test and Kruskal-Wallis test was performed for non-normally distributed variables. Spearman correlation coefficients were calculated to determine associations between changes in irisin and the changes in the biomarkers and anthropometric data (see Tables S1–S4 for complete results).

For the interventional arm of this study, we conducted a secondary analysis of the trial data, comparing the change in irisin from baseline to the 8-week visit between the treatment groups using an analysis of covariance model. The model included the percentage change from baseline as the dependent variable with adjustment for changes in fat mass and CRP over the eight week period. Percentage change from baseline was calculated as the difference between the eight week and baseline irisin levels divided by the baseline level. The model was also examined with 8-week irisin level as the dependent variable adjusted for the baseline irisin level, change in fat mass and change in CRP. Statistical significance was evaluated at an alpha level of 0.05 unless otherwise indicated. All statistical analyses were performed using SPSS Version 21 (SPSS, Inc., Armonk, NY). All data used in these analyses can be made available on request.

## Results

### Baseline Characteristics

The average age (±SD) of these 32 subjects was 41.4 (±13.8) with a BMI of 30.1 (±1.9). Other baseline characteristics of the study population are shown in Table 1. The three groups were not significantly different from each other aside from lower baseline CRP and IL-6 levels in the caffeinated coffee group. Of note, though age and gender distribution were similar in the 32 included and 9 excluded samples, the average BMI of the subjects analyzed was higher than that of the excluded samples (31.1 vs 27.9, p<0.001).

**Table 1:** Baseline characteristics of subjects by coffee group.

	Decaffeinated Coffee (n = 10)	Caffeinated Coffee (n = 12)	Placebo (n = 10)	p-value
Gender (% female)	3 (30)	5 (41.7)	5 (50)	0.70
Age (years)	40.3 (15.9)	36.7 (6.4)	48.3 (16.5)	0.14
Ethnicity (% non-Hispanic white)	8 (80)	6 (50)	7 (70)	1.00
BMI	30.1 (1.9)	29.7 (2.0)	30.6 (1.8)	0.56
Irisin (ng/mL)	55.4 (8.0)	47.8 (5.5)	50.6 (8.7)	0.07
Hip circumference (cm)	110.2 (4.2)	106.1 (6.4)	108.7 (7.4)	0.29
Waist circumference (cm)	105.5 (6.4)	96.9 (8.1)	101.1 (8.3)	0.05
Fat mass (kg)	29.5 (6.3)	25.1 (6.5)	29.3 (6.0)	0.19
CRP (mg/L)	3.3 (1.4–12.7)	1.0 (0.33–1.4)	1.8 (0.91–6.4)	0.04
IL6 (pg/mL)	2.0 (1.0)	1.1 (0.6)	2.3 (1.4)	0.04
Adiponectin (μg/mL)	6.6 (3.9)	6.2 (2.7)	7.2 (2.6)	0.75
FPG (mg/dL)	85.9 (12.9)	87.5 (9.6)	82.3 (11.7)	0.64
HOMA-IR	2.7 (1.4–4.2)	2.0 (1.1–2.8)	2.1 (1.5–2.4)	0.05

Significant difference between groups at p<0.05.

Data displayed as means (standard deviation) for continuous variables and number (percentage) for categorical variables.

CRP and HOMA-IR are shown as median (interquartile range).

Abbreviations: BMI, body mass index; CRP, C-reactive protein; IL-6, interleukin 6; FPG, fasting plasma glucose;

HOMA-IR, the homeostasis model assessment for insulin resistance.

### Correlation between Irisin Levels and Anthropometric Data

Correlations between irisin levels and baseline anthropometric data are shown in Table 2a. Baseline irisin levels were positively and significantly correlated with waist circumference (r = 0.41, p = 0.02) and fat mass (r = 0.44, p = 0.01) and this relationship remained even after adjustment for age and sex (r = 0.38, p = 0.04 for waist circumference and r = 0.43, p = 0.03 for fat mass, see table S1). Though there was a positive association between irisin and BMI (r = 0.25, p = 0.17), this did not reach significance.

**Table 2:** 

Table 2a. Spearman correlation coefficients between irisin levels and baseline anthropometric data							
	Baseline irisin (ng/mL)			% change in irisin		Absolute change in irisin (ng/mL)	
	r		p	r	p	r	p
BMI (kg/m^2^)	0.25		0.17	–0.15	0.41	–0.14	0.46
Waist circumference (cm)	**0.41**		0.02	–0.23	0.21	–0.23	0.22
Waist:Hip ratio	0.06		0.75	0.05	0.8	0.06	0.75
Fat mass (kg)	**0.44**		0.01	–0.33	0.07	–0.33	0.07
**Table 2b** **. Spearman correlation coefficients between irisin levels and changes in biomarkers**							
		**Baseline irisin (ng/mL)**		**% change in irisin**		**Absolute change in irisin (ng/mL)**	
		**r**	**p**	**r**	**p**	**r**	**p**
CRP (mg/L)		–0.12	0.51	**0.45**	0.009	**0.47**	0.007
IL6 (pg/mL)		–0.04	0.82	0.3	0.1	0.29	0.1
Adiponectin (μg/mL)		0.12	0.52	–0.11	0.54	–0.12	0.53
HOMA-IR		0.35	0.06	–0.006	0.97	–0.02	0.92
FPG (mg/dL)		0.11	0.56	0.18	0.35	0.18	0.35

Abbreviations: BMI, body mass index; CRP, C-reactive protein; IL-6, interleukin 6; HOMA-IR, the homeostasis model assessment for insulin resistance; FPG, fasting plasma glucose.

### Correlation between Irisin Levels and Other Biomarkers of Metabolism

Correlations between irisin levels and changes in other biomarkers over the study period are shown in Table 2b. Change in irisin positively correlated with change in CRP (r = 0.47, p = 0.007) though this was lost after adjustment for age, sex, and BMI (r = 0.31, p = 0.1, see table S4). Though these did not reach significance, irisin exhibited a direct relationship with change in IL6 (r = 0.29, p = 0.1) and a negative relationship with change in adiponectin (r = –0.12, p = 0.53) in the unadjusted model.

### Effect of Coffee Consumption on Irisin Levels

**Figure 2:**
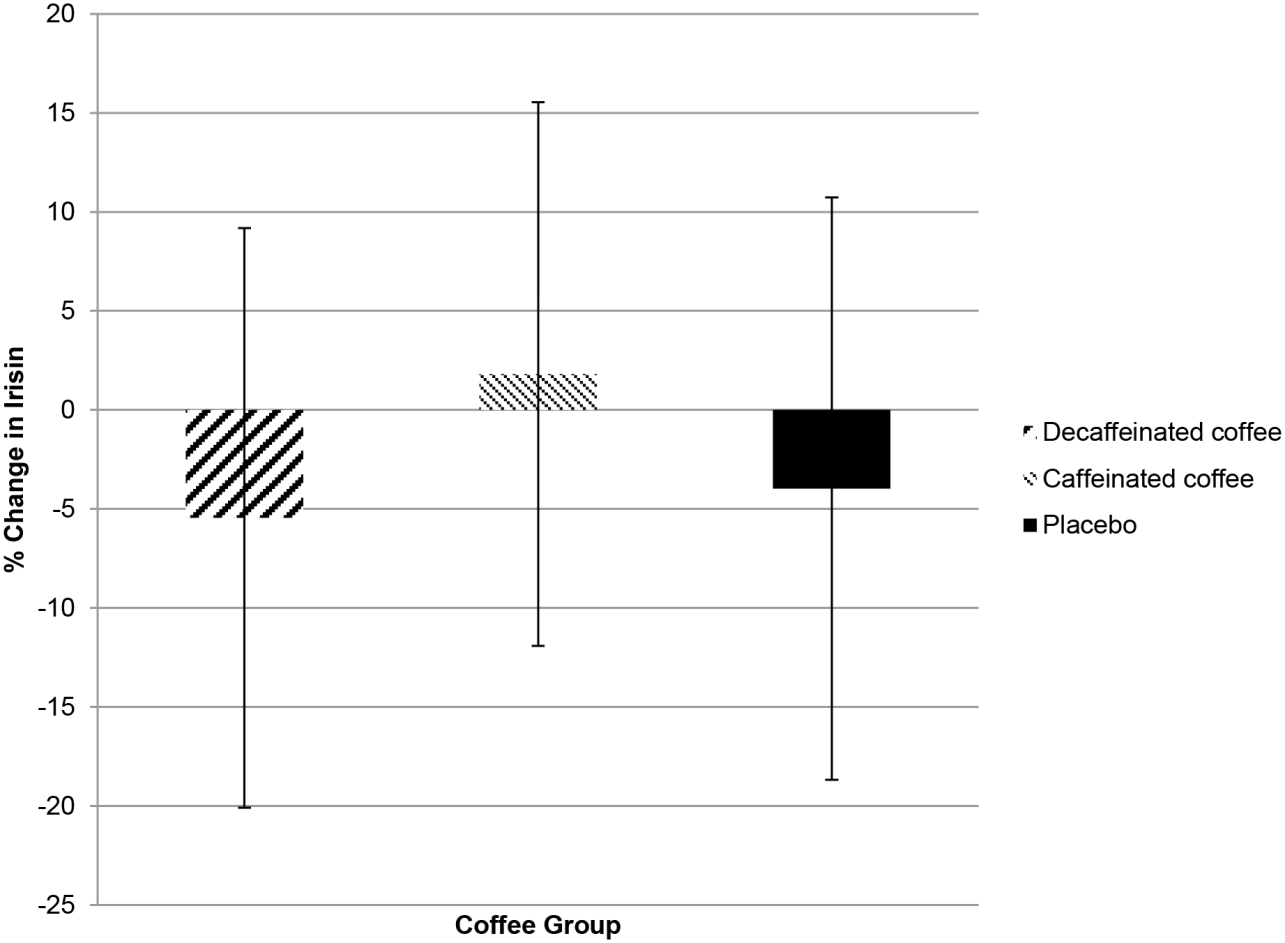
Percentage change in irisin levels over eight weeks by coffee group. Adjusted mean percent change estimates were determined from analysis of covariance models with treatment group as a main effect and changes in fat mass and CRP as covariates. 95% confidence intervals are graphically depicted.

Results of the ANCOVA model calculating percentage change in irisin levels over eight weeks by coffee group are graphically depicted in Figure 2. Over eight weeks, irisin levels decreased by 5.5% in the decaffeinated coffee group (95% confidence interval of −20.1 to 9.1) and by 4% in the control group (–18.7 to 10.7) while levels increased by 1.8% in the caffeinated coffee group (–11.9 to 15.5). Though irisin levels appeared to increase with caffeinated coffee consumption, this did not reach statistical significance (p = 0.75). The ANCOVA model using eight-week irisin level as the dependent variable with changes in CRP and fat mass as covariates also found no significant differences between the three groups (p = 0.83).

## Discussion

In this study, irisin levels demonstrated positive correlations with markers of adiposity such as fat mass and waist circumference and CRP, a marker of inflammation. While we had hypothesized that irisin levels would rise with increased caffeine consumption, this did not reach statistical significance.

The positive associations between irisin and markers of obesity in this study support the findings of several recent studies and are consistent with our prior hypothesis that irisin levels are increased in obesity as a means of counteracting rising insulin resistance [5], [8], [9], [11], [25]. A recent study found that irisin is secreted by adipose tissue in addition to skeletal muscle providing a mechanism that could explain the association between rising BMI and irisin levels [26]. In further support of this theory, though these associations failed to reach significance likely due to our small sample size, irisin tended to be positively associated with markers of inflammation and HOMA-IR and inversely related to the insulin-sensitizing hormone adiponectin. These findings add to the small but growing body of literature which suggests that irisin is associated with obesity-linked insulin resistance in humans. This continues to be an active area of research and more studies in humans are necessary to better understand the complex role of irisin in human metabolism.

Coffee intake increases energy expenditure and has been associated with a decreased incidence of diabetes and the metabolic syndrome in multiple studies [15], [16], [20]–[22]. The mechanism of this effect is not yet clear but could be a result of improved glucose uptake by skeletal muscle or up-regulation of insulin sensitizing hormones such as adiponectin [17], [20]. Though irisin is secreted in response to exercise, another state of increased energy expenditure, we did not detect a statistically significant change in irisin levels after eight weeks of coffee consumption [8]. Using these initial data, power calculations were performed using GPower 3.1.6 (Franz Faul, Universität Kiel, Germany) which showed that 53 samples would be required in each group to investigate this relationship, approximately five times the number analyzed in this cohort. Prior to this study there was no data with which to perform such a power analysis. Thus ultimately, a larger scale clinical trial would be needed to conclusively demonstrate whether the trend towards increased irisin levels in those who consume caffeine is indeed significant.

The key limitation of this study is the small and fixed sample size, limiting the power of the study to identify significant relationships and increasing the likelihood of extreme values affecting the results; however, prior to this study no data were available to determine the appropriate sample size. In terms of anthropometric data, the association between irisin and BMI might have been attenuated by the small range of BMI in this study given that all participants were overweight at baseline. The small size and relative homogeneity of this study population also limits generalizability of our findings.

Strengths of the study include that it is the first randomized controlled trial that has investigated the effects of coffee intake on irisin levels. We found positive associations between irisin and several markers of obesity, lending credence to the hypothesis that irisin may be secreted by fat in addition to muscle in response to obesity-linked insulin resistance. The data reported in this rather small study can be used to perform power calculations for larger clinical trials and mechanistic studies which are needed to more firmly establish the role of irisin in metabolism and to better understand its potential association with coffee.

## Supporting Information

Figure S1 **Scatter plot depicting irisin levels versus BMI and waist circumference.**
(TIF)

Figure S2 **Scatter plot depicting irisin levels versus waist: hip ratio and fat mass.**
(TIF)

Figure S3 **Scatter plot depicting irisin levels versus CRP and adiponectin.**
(TIF)

Figure S4 **Scatter plot depicting irisin levels versus fasting plasma glucose and HOMA-IR.**
(TIF)

Figure S5 **Scatter plot depicting irisin levels versus IL6.**
(TIF)

Table S1 **Spearman correlation coefficients between irisin levels and baseline anthropometric data.**
(TIF)

Table S2 **Spearman correlation coefficients between irisin levels and changes in anthropometric data.**
(TIF)

Table S3 **Spearman correlation coefficients between irisin levels and baseline biomarker levels.**
(TIF)

Table S4 **Spearman correlation coefficients between irisin levels and changes in biomarkers.**
(TIF)

Checklist S1 **CONSORT Checklist.**
(DOC)

Protocol S1 **Study Protocol.**
(DOC)
