# Supplementary figures and images for: Circulating Irisin Levels Are Not Affected by Coffee Intake: A Randomized Controlled Trial
Source: PLoS One. 2014 Apr 11;9(4):e94463. doi: 10.1371/journal.pone.0094463 (PMC3984159; doi:10.1371/journal.pone.0094463)

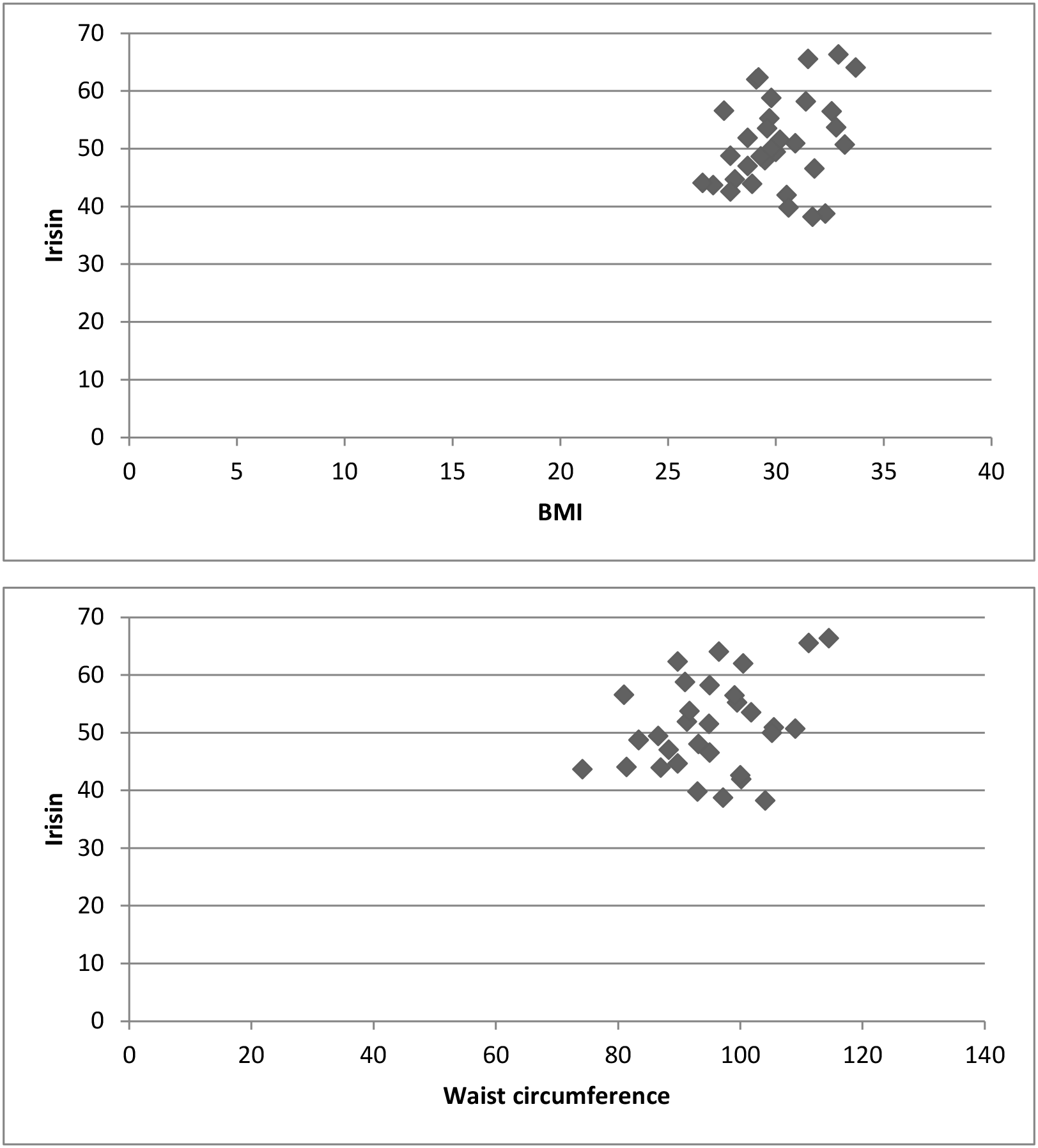

Supplement: Figure S1 — Scatter plot depicting irisin levels versus BMI and waist circumference. (TIF) [file pone.0094463.s001.tif]

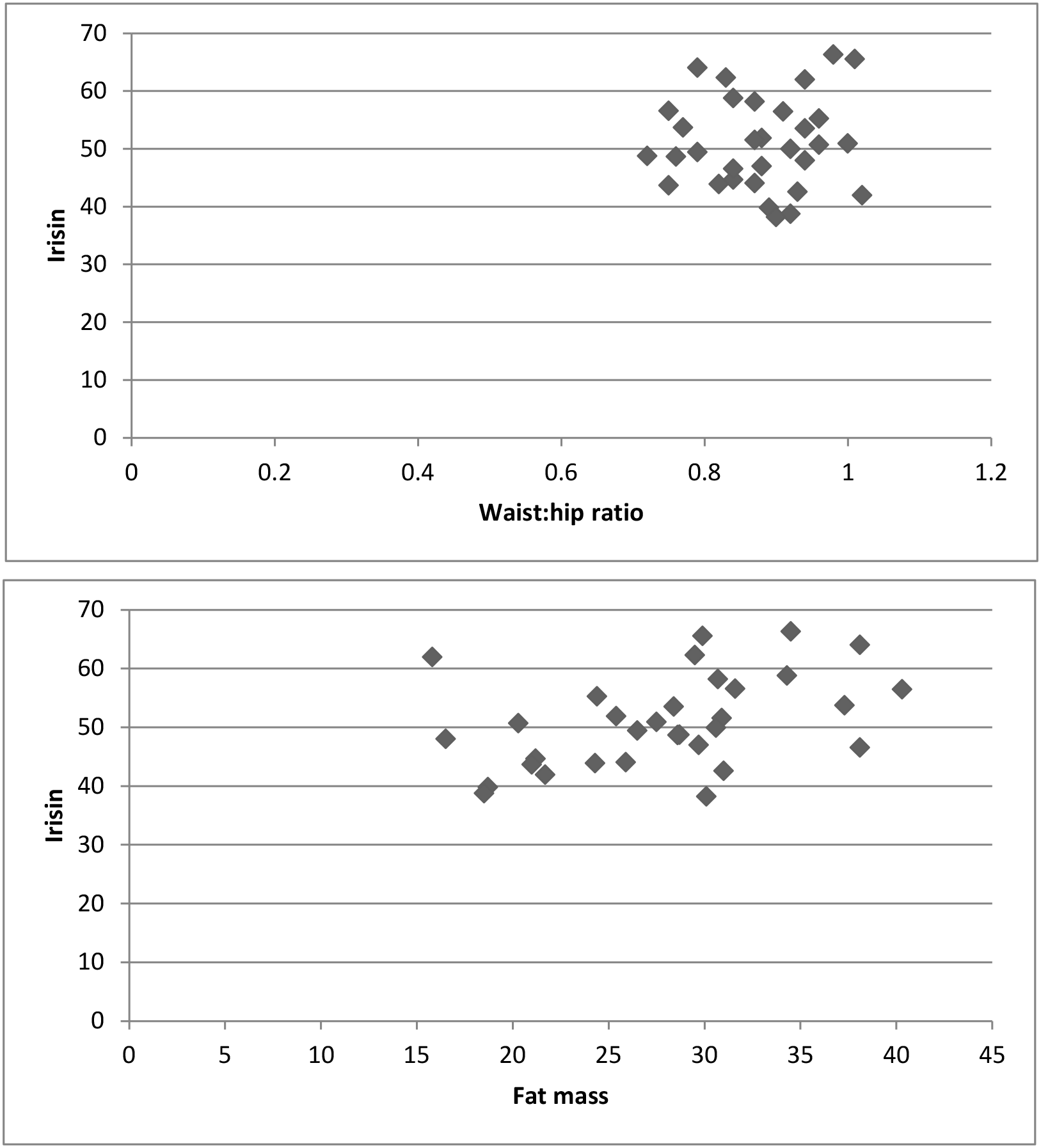

Supplement: Figure S2 — Scatter plot depicting irisin levels versus waist: hip ratio and fat mass. (TIF) [file pone.0094463.s002.tif]

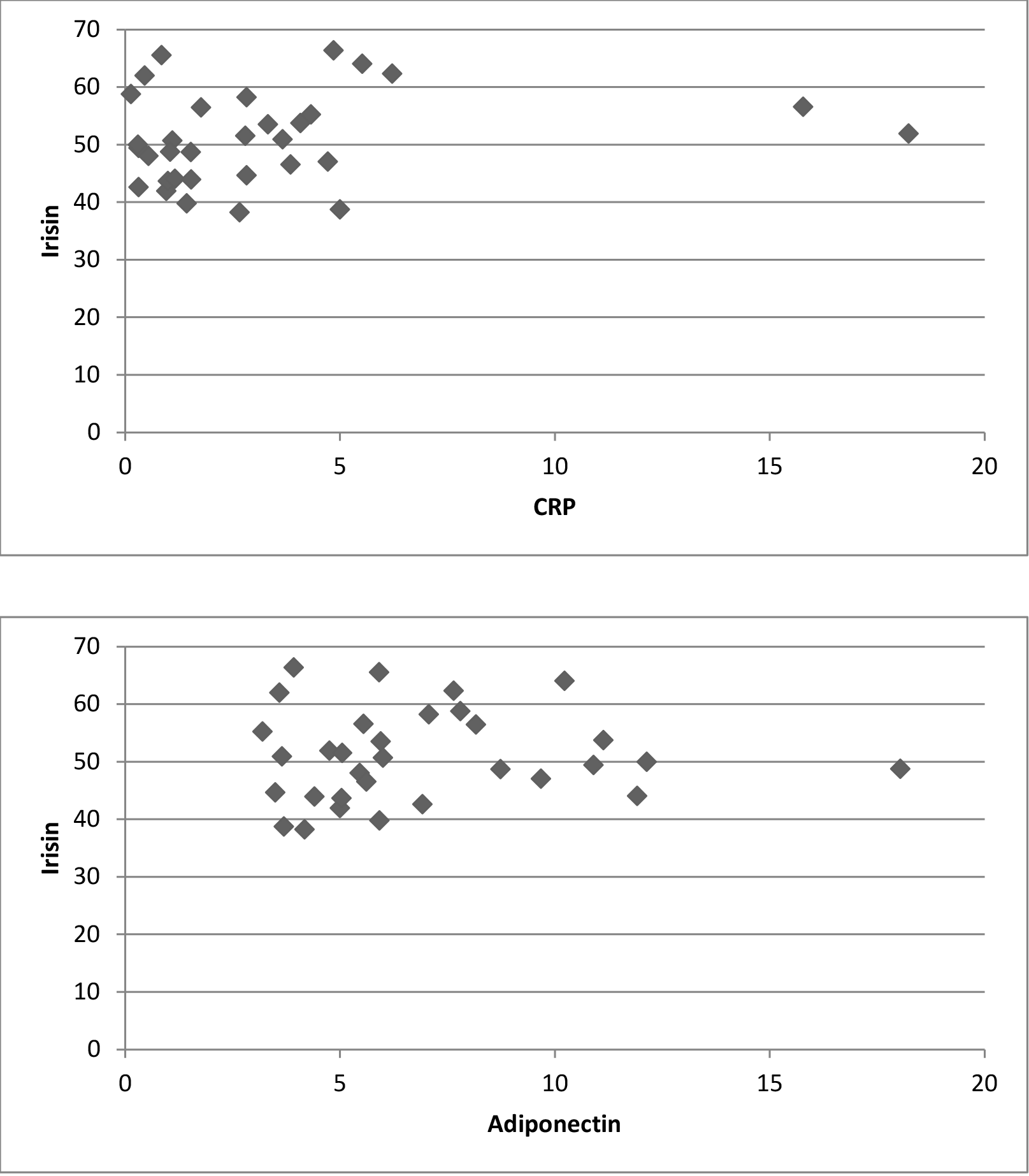

Supplement: Figure S3 — Scatter plot depicting irisin levels versus CRP and adiponectin. (TIF) [file pone.0094463.s003.tif]

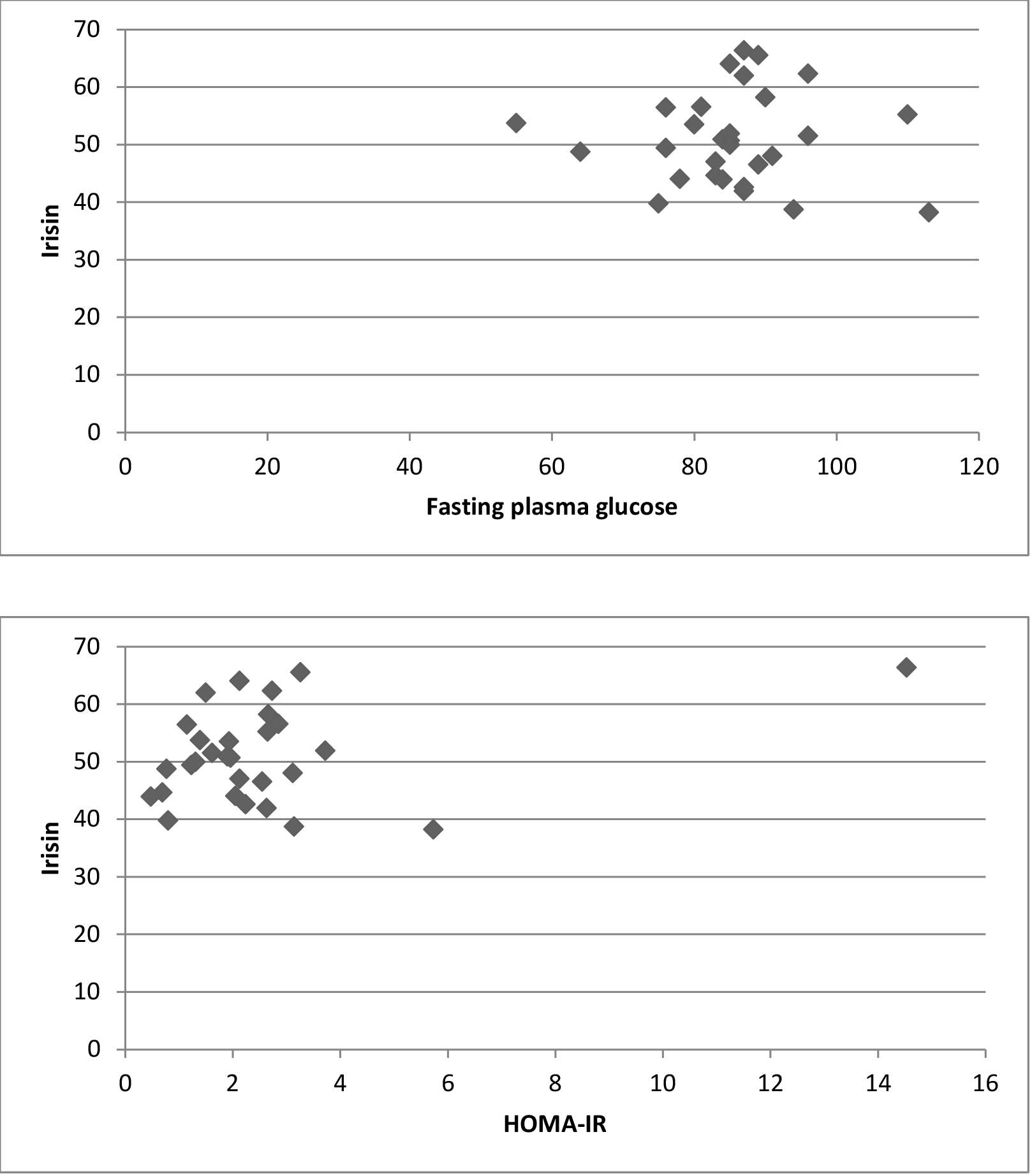

Supplement: Figure S4 — Scatter plot depicting irisin levels versus fasting plasma glucose and HOMA-IR. (TIF) [file pone.0094463.s004.tif]

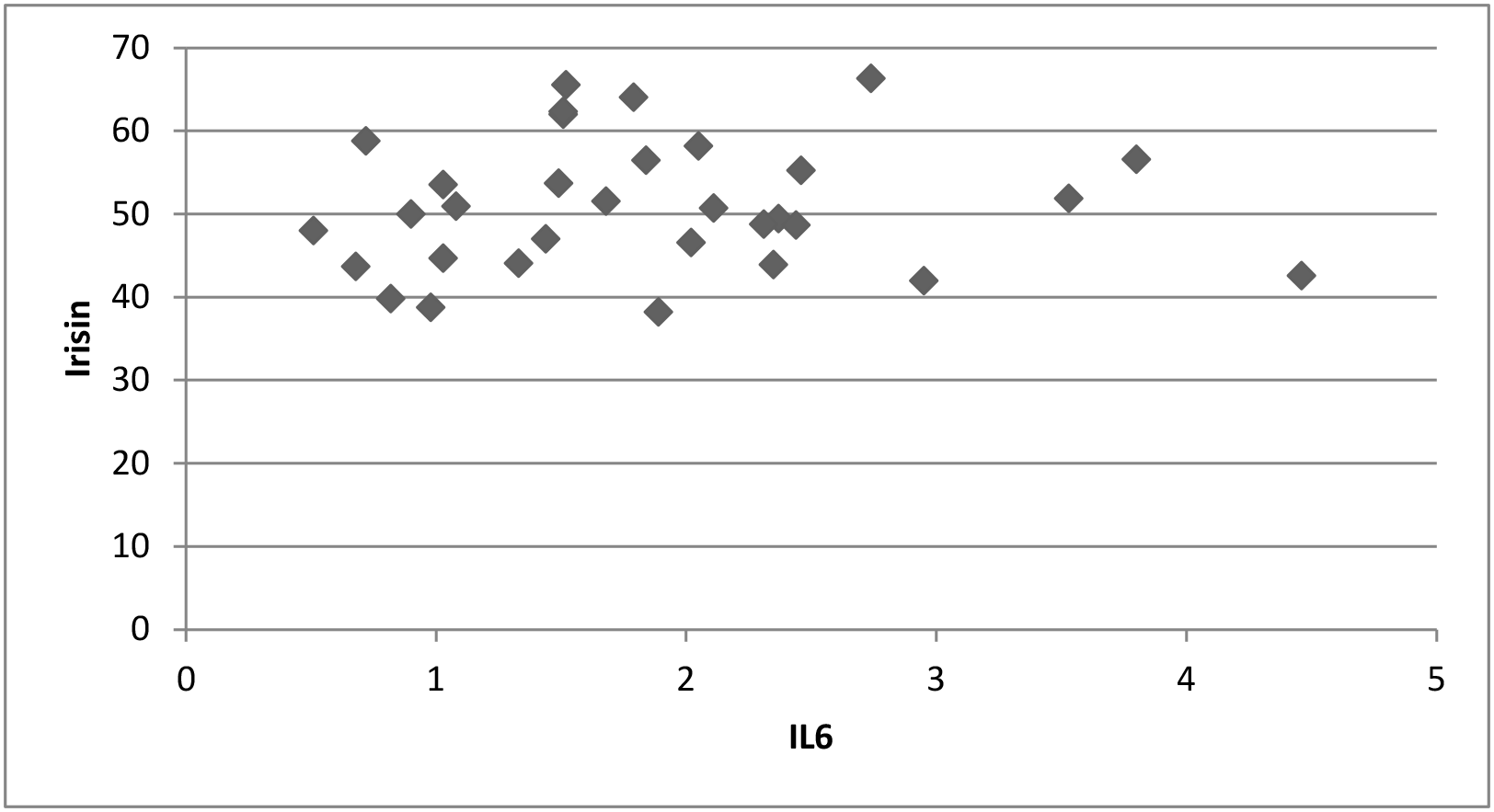

Supplement: Figure S5 — Scatter plot depicting irisin levels versus IL6. (TIF) [file pone.0094463.s005.tif]

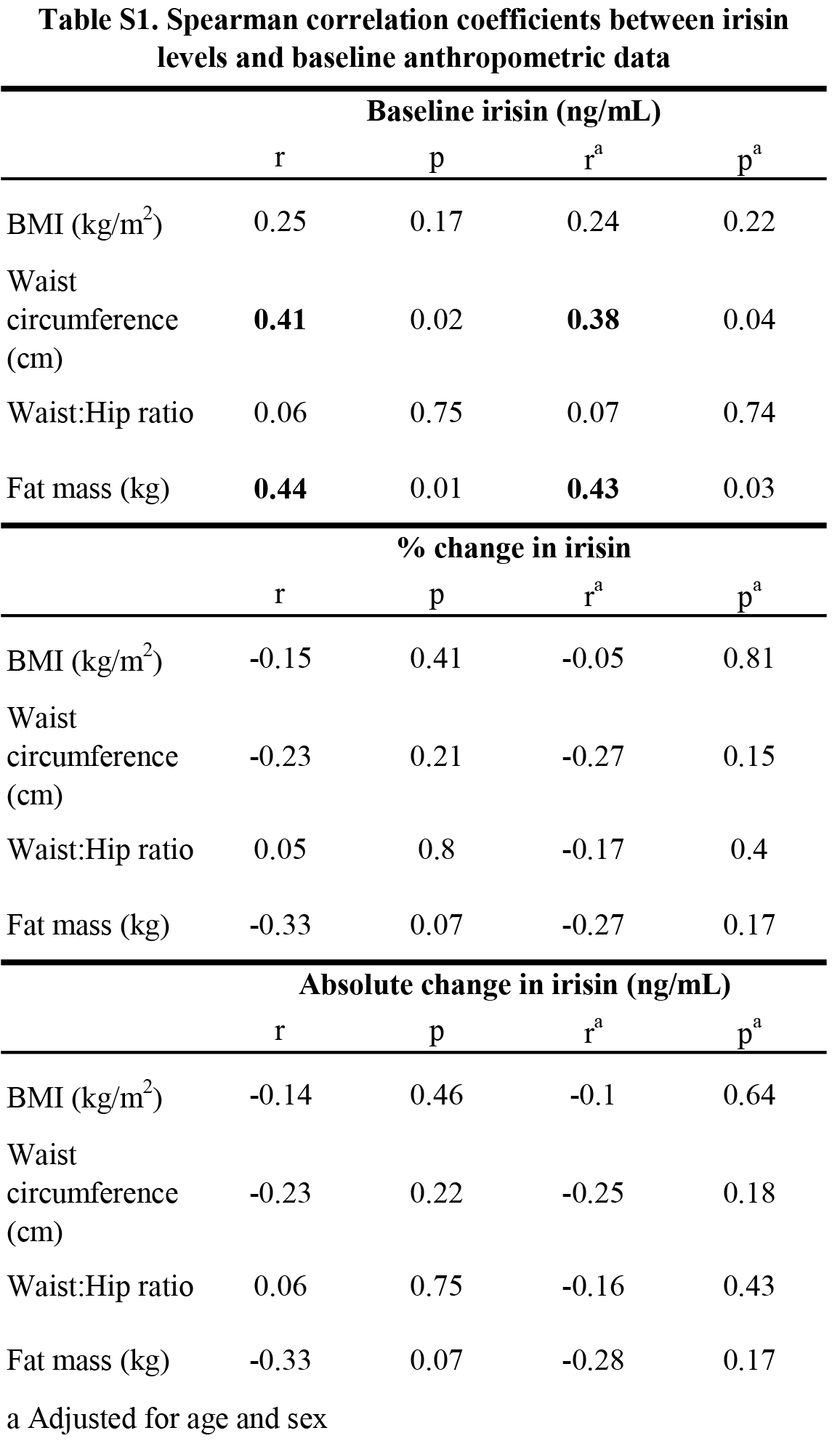

Supplement: Table S1 — Spearman correlation coefficients between irisin levels and baseline anthropometric data. (TIF) [file pone.0094463.s006.tif]

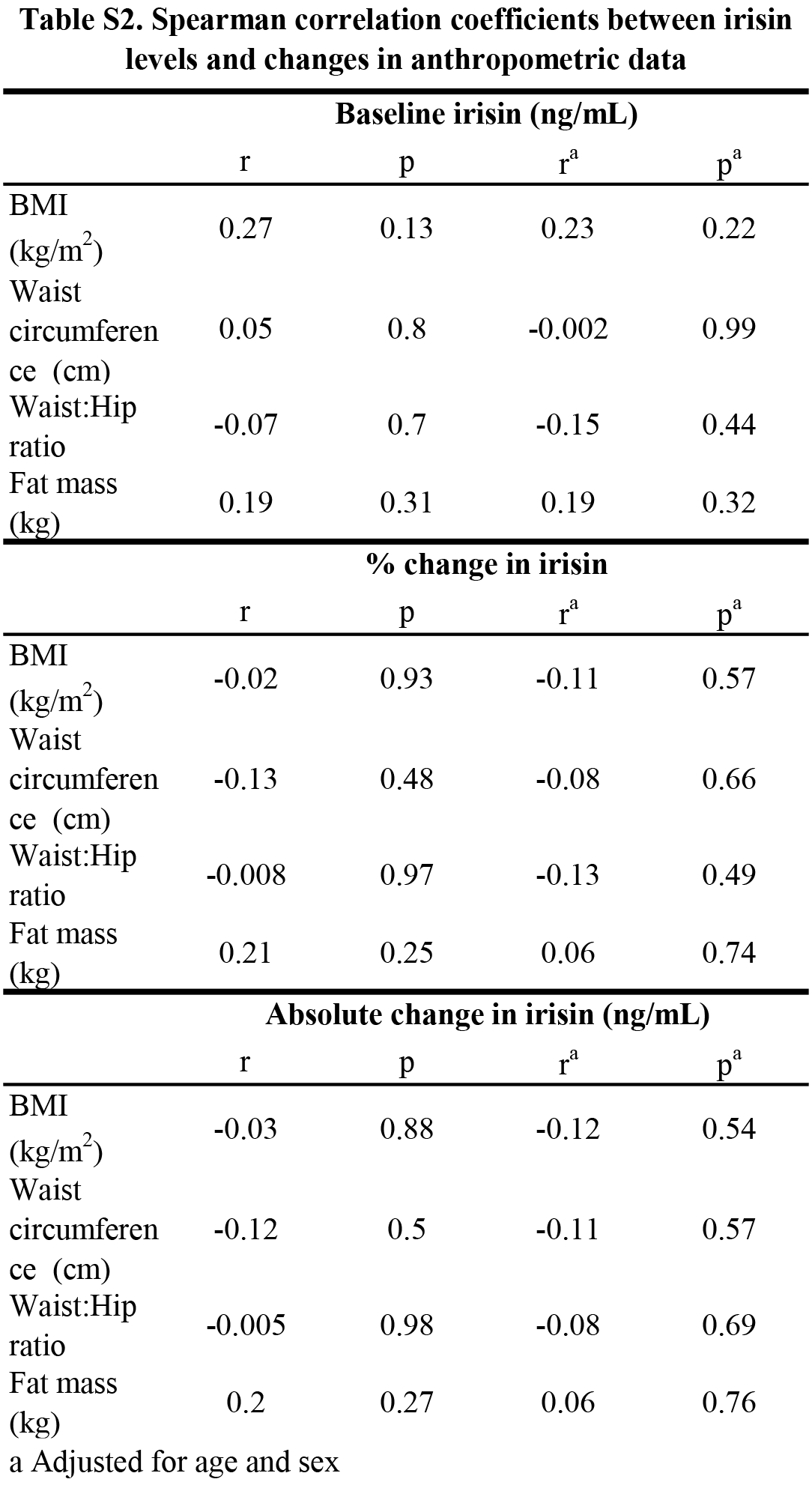

Supplement: Table S2 — Spearman correlation coefficients between irisin levels and changes in anthropometric data. (TIF) [file pone.0094463.s007.tif]

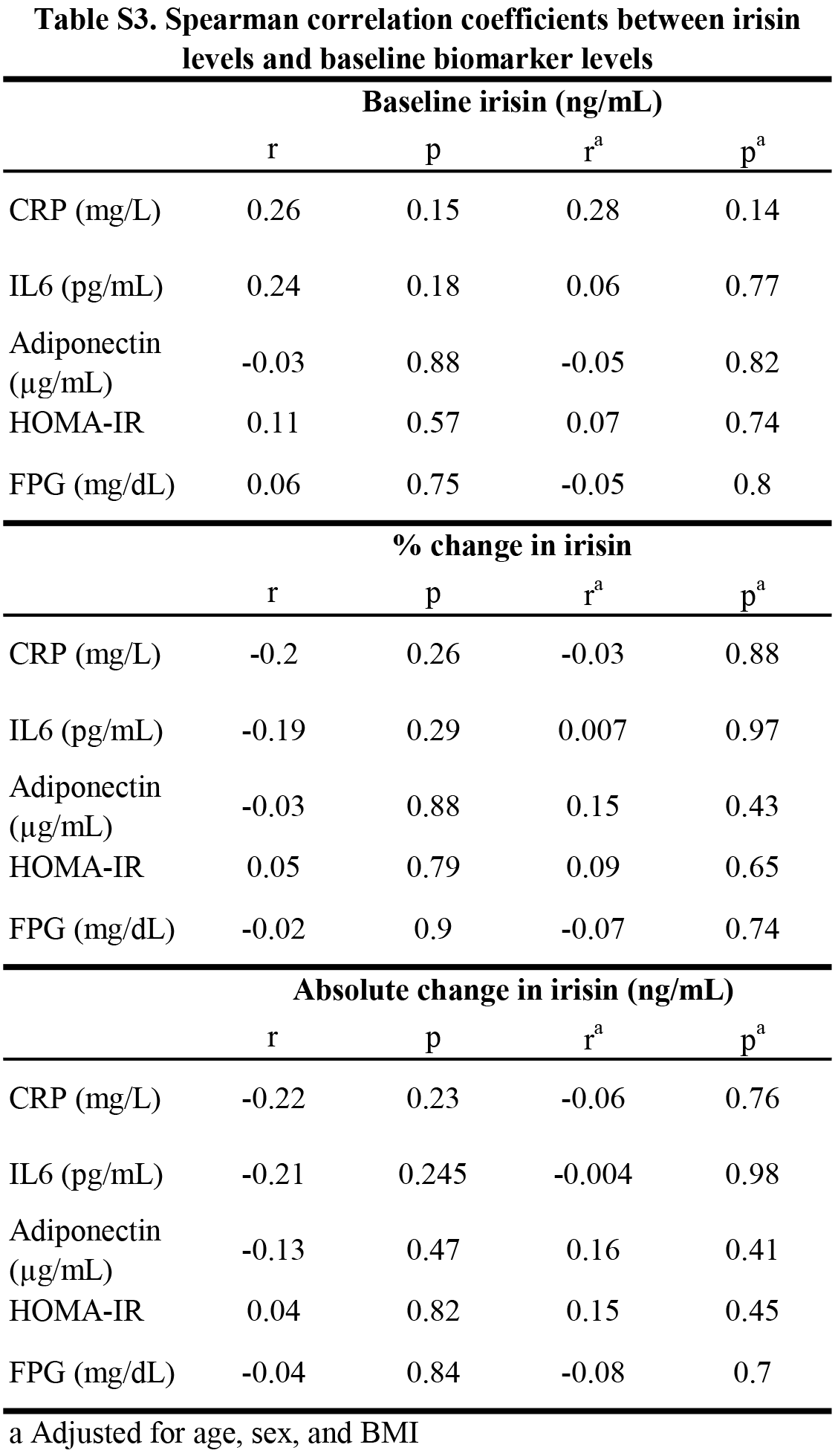

Supplement: Table S3 — Spearman correlation coefficients between irisin levels and baseline biomarker levels. (TIF) [file pone.0094463.s008.tif]

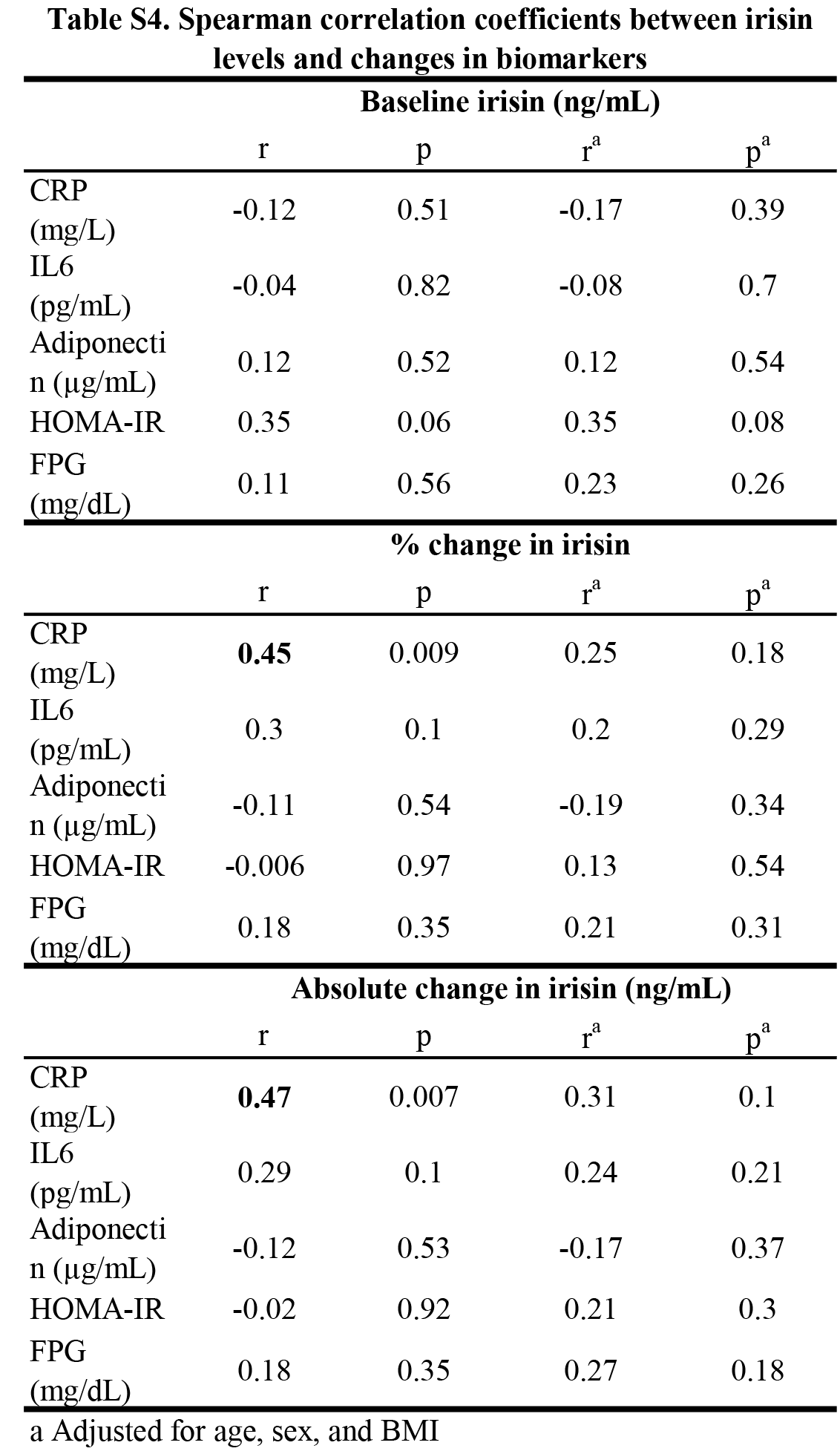

Supplement: Table S4 — Spearman correlation coefficients between irisin levels and changes in biomarkers. (TIF) [file pone.0094463.s009.tif]
