# Supplementary material for: Circulating Irisin Levels Are Not Affected by Coffee Intake: A Randomized Controlled Trial
Source: PLoS One. 2014 Apr 11;9(4):e94463. doi: 10.1371/journal.pone.0094463 (PMC3984159; doi:10.1371/journal.pone.0094463)
Supplement: Protocol S1 — Study Protocol. (DOC) [file pone.0094463.s011.doc]

# PART B

**STUDY DESCRIPTION**

| **Title of Protocol** | FORMTEXT Effects of caffeinated and decaffeinated coffee on body weight and glucose tolerance | | |
| --- | --- | --- | --- |
| Principal Investigator | Christos Mantzoros, MD | | |
| Co-Investigators | Rob M. van Dam, PhD, Aoife Brennan, MD, Frank B. Hu, PhD,  Qi Sun, Nicole Wedick | | |
| Mailing Address | BIDMC, 330 Brookline Ave., ST 816, Boston, MA | | |
| E-Mail Address | cmantzor@bidmc.harvard.edu | | |
| P.I.’s Telephone | 617-667-8633 | P.I.’s Pager 33086 | Fax: 617-667-8634 |
| Sponsor/Funding Source | Boston Obesity Nutrition Research Center | | |

# B1. PURPOSE OF PROTOCOL

| The overall aim of this clinical research proposal is to demonstrate the feasibility of randomized intervention study comparing the effects of consumption of caffeinated coffee, decaffeinated coffee or water on glucose metabolism. If successful, this study will form the basis for a long-term definitive trial of coffee consumption, body fatness, and glucose tolerance.  Specific aims of this study are:   1. To assess the adherence of randomized participants to their assigned treatment. 2. To determine the effect of caffeinated and decaffeinated coffee consumption on body weight, postload glucose concentrations and markers of insulin sensitivity. |
| --- |

#### B2. SIGNIFICANCE AND BACKGROUND FOR THE STUDY

| In the United States, 16 million persons, or almost 9% of adults, had diabetes in 2002 (1). Worldwide, an estimated 171 million persons have diabetes, and an alarming increase to 366 million persons is expected for the year 2030 (2). Type 2 diabetes accounts for 90-95% of all diagnosed cases of diabetes. Diabetes is associated with high rates of morbidity and premature mortality. Obesity increases risk of type 2 diabetes, hypertension, arthritis, cardiovascular diseases, several types of cancer, and premature mortality (3).  While increased physical activity and restriction of energy intake can substantially reduce incidence of type 2 diabetes (4) insight into the role of other lifestyle factors may contribute to additional prevention strategies for type 2 diabetes. Habitual consumption of coffee may have substantial beneficial effects on glucose metabolism according to recent findings of epidemiological studies in the U.S., Europe, and Japan (5). Coffee is among the most widely consumed beverages in the world. Knowledge on both the positive and negative health effects of coffee is important to allow individuals to make informed choices regarding coffee consumption. In addition, data on the health effects of different types of coffee can be used in public health recommendations and may contribute to disease prevention.  Coffee in relation to glucose metabolism and energy balance: potential mechanisms  Coffee is a beverage that contains hundreds of components including substantial amounts of chlorogenic acid, caffeine, magnesium, potassium, niacin (vitamin B3), trigonelline (N-methylnicotinic acid), and lignans (6-9). The table shows a selection of coffee components and suggested mechanisms for effects of these components on glucose metabolism. Most data on effects of coffee components on glucose metabolism are based on animal and *in vitro* studies, and the relevance for the weight gain and the development of type 2 diabetes in humans is currently unclear. However, the results suggest that an effect of coffee consumption on body fatness and glucose metabolism is biologically plausible, and that the effects of coffee cannot be equated with those of caffeine.  *Caffeine:* caffeine intake was associated with an acute reduction of insulin sensitivity in short-term metabolic studies in humans (10,11) This effect reflects decreased glucose storage, probably due to increased epinephrine release (11) Acute effects of caffeine through increased epinephrine levels cannot be extrapolated to long-term effects of coffee consumption, because acute effects of coffee on epinephrine are weaker than expected from its caffeine content (22) and because effects of caffeine on epinephrine levels wane after continued intake (23) Based on animal studies and metabolic studies in humans, beneficial effects of caffeine on glucose metabolism through increased uncoupling protein expression and lipid oxidation have also been suggested (12). Recent findings from observational studies indicate that the inverse association between coffee consumption and risk of type 2 diabetes cannot be explained caffeine. Decaffeinated coffee consumption was also associated with a lower risk of type 2 diabetes and with markers of insulin sensitivity (24,25). These results indicate that components of coffee other than caffeine can affect glucose metabolism, but do not rule out an effect of caffeine on energy balance and glucose metabolism. In rats, caffeine injections reduced energy intake and weight gain, an effect that may have been mediated by increased levels of corticotrophin-releasing factors or sympathetic activation (16). In humans, energy intake was increased during 2 days of caffeine withdrawal (26). In addition, short-term intervention studies have consistently shown that intake of caffeine or caffeinated coffee stimulates thermogenesis and increases energy expenditure (13-15).  Epidemiological studies on coffee consumption and risk of type 2 diabetes  We have recently conducted a systematic review on epidemiological studies on coffee consumption and risk of type 2 diabetes (5). Nine cohort studies on coffee consumption and risk of type 2 diabetes included a total of 193,437 participants and 8394 incident cases of type 2 diabetes. The summary multivariate-adjusted relative risk of type 2 diabetes was 0.65 (95% CI 0.54-0.78) for the highest (≥6 or ≥7 cups per day) and 0.72 (95% CI 0.62-0.83) for the second highest (4-6 cups per day) category of coffee consumption compared with the lowest category of coffee consumption (0 or ≤2 cups per day). In a recent prospective U.S. study, that was not yet included in the meta-analysis, coffee consumption was also inversely associated with risk of type 2 diabetes (27). In cross-sectional studies, coffee consumption was consistently associated with a lower prevalence of impaired glucose tolerance and type 2 diabetes (5). In several, but not all studies, higher habitual coffee consumption was associated with higher insulin sensitivity (5,28). Results from studies that included an oral glucose tolerance test suggested that coffee consumption affected postprandial glucose metabolism, rather than fasting glucose concentrations (5,29). Coffee consumption tends to be associated with lifestyle habits that increase risk of type 2 diabetes (e.g. cigarette smoking, lower physical activity) and more complete adjustment for confounding generally strengthened the observed inverse association between coffee consumption and risk of type 2 diabetes (5). Still, confirmation of the observed inverse association between coffee consumption and risk of type 2 diabetes in randomized trials of coffee consumption with appropriate outcome measures is desirable to completely rule out residual confounding as a potential explanation.    Previous intervention studies  Data from intervention studies on the effect of coffee consumption for longer than 1 day on glucose homeostasis is sparse. In an older study, 14 days of higher decaffeinated coffee consumption was associated with a decrease in plasma glucose concentrations (30). However, this intervention study did not include a control group. In a randomized cross-over study, 4 weeks of very high coffee consumption did not affect fasting glucose concentrations and increased fasting insulin concentration (31). This increase may reflect reduced hepatic extraction of insulin or direct effects of caffeine on insulin secretion,(31) and requires further study. Several studies have tested effects of caffeine intake of glucose levels and observed no effects (5). No randomized intervention studies have examined the effects of coffee consumption for longer than 1 day on postprandial glucose levels. This is of particular importance because coffee consumption was associated with lower postload (during an oral glucose tolerance test), rather than lower fasting glucose concentrations (5). Few studies have examined the effect of caffeine intake on weight loss. Intake of caffeine capsules for 24 weeks did not induce weight loss in obese persons (32). However, in an other study overweight and moderately obese persons who were habitual caffeine consumers lost more weight during a dietary intervention than those that did not consume caffeine (33).  Study significance:  Epidemiological studies have consistently shown an association between higher coffee consumption and a lower risk of type 2 diabetes. Confirmation of these findings in randomized trials of coffee consumption is desirable, and no randomized intervention studies of coffee consumption and glucose tolerance lasting longer then 1 day has been conducted. We propose this pilot study to demonstrate the feasibility of such a trial. If successful, this study will form the basis for a definitive trial of coffee consumption, body fatness, and glucose tolerance. Given the extensive use of coffee and the rapidly increasing health burden of type 2 diabetes and other obesity related disorders, such a trial would have important public health implications.  Preliminary Data: We have not yet conducted a coffee consumption trial, but an Australian trial of caffeinated and decaffeinated coffee protocol similar to that in the current study appeared to be well tolerated by participants with satisfactory compliance (34). |
| --- |

#### B3. DESCRIPTION OF RESEARCH PROTOCOL

##### A. Study Design

| For this pilot trial we will use an 8-week randomized intervention study comparing the effects of caffeinated coffee (n=15), decaffeinated coffee (n=15), and no coffee (n=15) consumption (see figure).  the study medications. The 8-week intervention will be preceded by a 2-week run-in phase with no coffee consumption. At the initial visit, the 4 week visit, and the 8 week visit an oral glucose tolerance test (OGTT) will be conducted and measures of body fatness will be assessed. At the 6-week visit a nonfasting blood sample will be obtained to measure caffeine concentrations. Although we recognize the potential advantages of a cross-over study, the compliance to the interventions is uncertain and a cross-over design is particularly sensitive to decreases in compliance over time. The intervention will be blinded to the investigators. The intervention will be double blind with regard to the coffee type that participants receive (caffeinated and decaffeinated); we will ask the participants about whether they notice the type of coffee they receive.  **Screening** **Initial visit** **4 wk visit 6 wk visit** **8 wk visit**   | Run-in-phase (2 weeks)  (No coffee) | - Caffeinated coffee (n=15) - Decaffeinated coffee (n=15) - No coffee (n=15) | | --- | --- |   **Randomization:**  Randomization will be conducted by a statistician that is not involved in the study using blocking and stratification by sex to avoid differences in the proportion of men and women between the three intervention groups (35).  **Interventions:**  Participants in the intervention groups will consume 5 cups of instant caffeinated or decaffeinated coffee. The instant coffee will be provided in prepackaged plastic cups each containing the same amount of coffee that should be used to prepare one cup of coffee by adding hot water and 5 of these plastic cups are included in 1 bag (supply for 1 day). The 1-cup packages will not provide any clues whether they contain caffeinated or decaffeinated coffee. We will instruct participants on how to prepare the coffee, and ask them to consume a cup with every meal, and at mid-morning and mid-afternoon. Participants will be allowed to adjust the amount of added water according to their preferred coffee strength, but coffee should be consumed without caloric sweeteners. Artificial sweetener will be supplied to participants that prefer sweetened coffee. Participants who are randomized to the control (no coffee) intervention will be instructed to consume 5 cups of water. Before the start of the run-in phase, participants will receive dietary advice from a nutritionist to avoid coffee-related and caffeine-containing foods and drinks other than the coffee that will be provided. They will also be instructed to maintain a stable usual diet (including supplement use and alcohol consumption), and stable physical activity patterns throughout the study. In addition, participants are not allowed to use caffeine-containing medications such as certain flu remedies and analgesics or medications that may interact with caffeine including bronchodilators, quinolone antibiotics, monoamine oxidase inhibitors, anxiolytics, ranitidine, corticosteroids, growth hormone, and antihypertensives. To reduce symptoms related to caffeine withdrawal, we will instruct participants to gradually reduce their coffee consumption at the start of the run-in period and after completion of the trial over three days. We will provide extra coffee to the participants to allow this gradual reduction after completion of the trial.  **Visits**  Visits will be arranged as outpatient visits to the GCRC and none of the visits will not require an overnight stay.  1. Screening visit (1 visit)  The screening visit consists of:   - Signing the informed consent - An interview: medical history, medication use, use of drugs and alcohol, habitual physical activity - A physical examination including measurement of weight and height - An electrocardiogram, a urine HCG pregnancy test (women only), and drawing of fasting blood for blood glucose, liver function tests (AST, ALT, alkaline phosphatase), creatinine, and complete blood count (CBC).   2. Consultation nutritionist and run-in period  Subjects will meet with the nutritionist to receive instructions to avoid coffee-related and caffeine-containing food (other than the coffee provided) during the entire study. They will also be instructed to maintain a stable usual diet, and stable physical activity patterns throughout the study. To document adherence with the diet, subjects will be asked to record the type of foods and the food portions eaten for two weekdays and one weekend day for the week before the post-run-in phase visit and the visits 4 and 8 weeks later. Following this consultation, subjects will enter the 2-week run-in phase, which will serve as a washout period for the effects of previous coffee/caffeine use.  3. Post run-in phase and efficacy assessment visits (three visits)  These visits will occur immediately after the run-in-phase and 4 and 8 weeks after that. During these visits we will:   - Measure weight and waist circumference - Assess body composition using bioelectrical impedance analysis. - Measure systolic and diastolic blood pressure. - Collect fasting blood samples and conduct an oral glucose tolerance test drawing blood at 30 min, 60 min, and 120 min after the oral glucose load. Blood samples will be used to measure glucose, insulin concentrations, and lipid profile (LDL, HDL, and total cholesterol, triglycerides) and will be stored for possible future measurement of intermediary factors (e.g. adipokines). - Supply coffee sachets to the participants and provide instructions on how to prepare the coffee ( post run-in phase visit and week 4 visit only) - Count what is left of the provided coffee sachets to assess compliance (week 4 and week 8 visit only). Subjects will be invited to provide their ideas regarding possibilities to improve compliance. - Collect the 3-day dietary records and diaries in which participants will document deviations from the intervention protocol.   4. Visit with nonfasting blood draw (one visit)  This visits will occur in the afternoon 6 weeks after the run-in-phase. During this visit we will measure weight and systolic and diastolic blood pressure and collect a nonfasting blood sample. This sample will be used to measure s**erum caffeine concentrations to assess compliance.**  **Consistency of the provided coffee**  We will obtain instant coffee that is from the same batch to reduce variation in coffee characteristics. Also, we will store the product under optimal conditions (e.g. closed containers) and we will measure the amount of caffeine in caffeinated and decaffeinated coffee samples obtained at the start (that will be frozen) and at the end of the study.  **Technology employed in the study**  Demographic data and medical history: obtained by using a standardized questionnaire.  Anthropometry: Body weight, height and waist circumference will be measured according to standard protocols. The body mass index (BMI) is calculated as the weight (kg) over height^2^ (meters squared). Body composition will also be assessed by bioelectrical impedance analysis, using a single frequency bioimpedance analyzer (model 101, RJL Systems, Mt. Clemens, MI), and fat-free mass (FFM) and fat mass (FM) will be calculated (36). This method has previously been validated by comparison with DEXA measured FM (r=0.91) (37).  Dietary data: Using 3-day dietary records (2 weekday and 1 weekend day) we will calculate total energy intake, the number and distribution of meals and snacks, macro-and micronutrient intakes, and consumption of foods and drinks (particularly carbonated beverages and dairy using the Nutritionist Five program (First Data Bank, San Bruno, CA).  Assessment of blood pressure: blood pressure will be measured using multiple repeated measured with automatic Dinamap blood pressure meters using measurements after the participants has been seated for at least 5 minutes.  Oral glucose tolerance test (OGTT): An OGTT will be conducted in the morning starting before 10 am. Subjects will be instructed not to eat or drink after 8 pm not to use coffee after 5 pm on the preceding day. The glucose load will consist of 75 g glucose anhydrate in 300 ml of water ingested over the course of 5 min. An intravenous catheter will be placed in the forearm and blood samples will be collected before the oral glucose load (fasting), after 30 min, 60 min, and 120 min.  Laboratory data: Insulin will be measured using ELISA without cross-reactivity for pro-insulin, glucose will be measured using a photometric method. Fasting triglycerides and lipoprotein profile will be measured in a certified clinical lipid lab using standard methods (38,39). **Serum caffeine concentrations will be measured (using HPLC) in nonfasting blood samples obtained at the 6-week visit and in the coffee samples. B**lood samples will be stored for future determination of other possible intermediary effects of coffee using other funding sources.  Assessment glucose tolerance and insulin resistance: We will use the glucose area under the curve and 2-hr glucose as measures of glucose tolerance. Higher coffee consumption was associated with lower 2-hr glucose concentrations during an oral glucose tolerance test in several cross-sectional studies (5,29). We will calculate the ratio between the 30 min increment in insulin to 30 min increment in glucose concentration as a measure of early insulin secretion (40). We will calculate the homeostasis model assessment of insulin resistance calculated using the following formula: insulin resistance = fasting insulin x glucose / 22.5 (41). In addition, we will calculate the composite insulin sensitivity index (CISI) using the formula {10,000/the square root of [(fasting glucose x fasting insulin) x (mean glucose x mean insulin)]} during the oral glucose tolerance test. This measure has been shown in validation studies to provide a reasonably good measure of insulin resistance (42). |
| --- | --- | --- |

### B. Statistical Considerations

| SAS 8.2 (SAS Institute Inc., NC, USA) will be used for statistical analyses. All safety and adverse event data will be summarized separately for each group. The statistical analysis of the data will follow a standard intention to treat analysis. Changes in glucose and insulin concentrations, weight, body mass index (kg/m^2^), waist circumference, calculated FFM and FM and dietary intake will be evaluated. We will test overall time effects, treatment effects, and time*treatment interactions using ANOVA, and compare the changes in outcome measures between the groups using Student t-tests (if necessary after normalization of the outcome variable, or using the Wilcoxon Rank Sum test). First analyses will be conducted separately for changes between 0 and 4 weeks and for changes between 0 and 8 weeks. If similar results are obtained, the change between 0 weeks and the average of 4 and 8 weeks can be tested to reduce random within person variation. We will also conduct a data analysis stratifying by habitual coffee consumption before the start of the trial (i.e. 1-2 cups, 2-4 cups and >4 cups per day).  A goal of this pilot study is to obtain information on differences in outcome measures and variation to be used in a power analysis for a ‘definite’ trial which will be proposed in a future R01. The available data is insufficient to conduct a power analysis for effects of coffee on the outcome measures at this point, but previous dietary intervention studies of smaller size and shorter duration have been able to detect effects on parameters of glucose metabolism (43). |
| --- |

### C. Subject Selection

| Overweight adult men and women will be recruited from the Boston community through advertisement and postings without regard for racial, social, economic, or other status.  *Inclusion criteria:*   1. Aged at least 18 years with an ability and willingness to give written informed consent. 2. Body mass index 25-35 kg/m^2^ 3. Users of at least 2 cups of caffeinated coffee per day who are willing to be randomized to any of the interventions. 4. Non-smoking   *Exclusion criteria:*   1. Any condition/illness that may affect the study outcomes or would make participation potentially harmful such as pregnancy or breastfeeding, diabetes mellitus, heart disease, stroke, hypertension, malabsorption syndromes, GERD, a history of ulcer, according to a detailed medical history. 2. Abnormal hepatic function (liver function test > twice the normal range), abnormal renal function (creatinine > 1.1 mg/dl), fasting plasma glucose in the diabetic range (>/= 126 mg/dl), or blood pressure > 140/90 mmHg. 3. Present alcoholism or drug abuse or use of medications that could interfere with the treatment including bronchodilators, quinolone antibiotics, monoamine oxidase inhibitors, anxiolytics, ranitidine, corticosteroids, growth hormone, antihypertensives. These conditions will be screened for by a detailed history and systems review. |
| --- |

#### B4. POSSIBLE BENEFITS

| Subjects will receive a free screening, body composition and evaluation of glucose metabolism. It is hoped that the information from this study eventually will contribute to preventive efforts with regard to risk of type 2 diabetes. |
| --- |

#### B5. POSSIBLE RISKS AND ANALYSIS OF RISK/BENEFIT RATIO

| Risks to the subjects will be minimal, including slight pain, or bruise at the site of venipuncture. Potential side effects of the coffee use and caffeine withdrawal will be monitored closely. Caffeine abstinence can lead to withdrawal symptoms in regular caffeine users including headaches, fatigue, decreased energy/ activeness, decreased alertness, drowsiness, decreased contentedness, depressed mood, difficulty concentrating, irritability, being foggy/not clearheaded, flu-like symptoms, nausea/vomiting, and muscle pain/stiffness (44). Typically, symptoms of caffeine withdrawal have a peak intensity after 1-2 days and have a duration of 2-9 days (44). Increases in homocysteine concentrations (45) and blood pressure (average increase: 1.2 mmHg for systolic and 0.5 mmHg for diastolic blood pressure) (46) have been reported as a results of (caffeinated) coffee consumption and systolic and diastolic blood pressure will be measured at each visit. If any serious side effects of the interventions are detected, participants will be withdrawn from the study protocol.  Risk protection / prevention procedures  We will ask the subjects to stay in close communication with us in case any symptoms that could possibly be attributed to the study. One of the investigators will carry a pager at all times for 24 hour per day access by subjects. Confidentiality will be maintained through our computer‑based data coding system. Results obtained from individual subjects will be analyzed, reported and discussed in reference to the subjects code. The principal investigator will maintain complete control over all data. If there appears to be good reason to share information with other investigators this will be done only by reference to the subject's entry code. |
| --- |

#### B6. RECRUITMENT AND CONSENT PROCEDURES

| Recruitment Strategy  We plan to enroll ~45 subjects over 4 weeks. To achieve this, we will need to screen approximately 20-25 subjects per week at the GCRC (to have 1 subject meeting the study eligibility criteria out of approximately 2 subjects screened). We expect that the number of persons that has to visit the hospital for screening will be relatively low because the eligibility criteria are not very restrictive and can to a large extent be assessed through a preceding telephone interview. We will recruit from the Boston community through advertisements and posters. We have extensive experience with recruiting and screening subjects and have previously used successfully the techniques we propose to use herein. Thus, we do not feel that enrolling the number of patients needed would pose any problems.  Consent procedures  The purpose of the study, the risks involved, reimbursements for study related costs, and the importance of the subject's full cooperation will be explained to each subject and will also be included in the consent document, which each subject will read and sign before entering the study. Details of the protocol will be explained to subjects and written informed consent will be obtained. The subjects also will be informed that they may withdraw from the study at any time. Constant medical supervision will be provided throughout the study period. A copy of the protocol and the consent form will be given to each patient and a copy of the consent form will be archived. |
| --- |

## B7. STUDY LOCATION

| BIDMC General Clinical Research Center. |
| --- |

**References:**

1. Centers for Disease Control and Prevention. National diabetes fact sheet: general information and national estimates on diabetes in the United States, 2002. Atlanta, GA: U.S. Department of Health and Human Services, Centers for Disease Control and Prevention, 2003.

2. Wild S, Roglic G, Green A, Sicree R, King H. Global prevalence of diabetes: estimates for the year 2000 and projections for 2030. Diabetes Care 2004; 27:1047-53.

3. Willett WC, Dietz WH, Colditz GA. Guidelines for healthy weight. N Engl J Med 1999; 341:427-34.

4. Knowler WC, Barrett-Connor E, Fowler SE, et al. Reduction in the incidence of type 2 diabetes with lifestyle intervention or metformin. N Engl J Med 2002; 346:393-403.

5. Van Dam RM, Hu FB. Coffee consumption and risk of type 2 diabetes. A systematic review. JAMA 2005;294:97-104.

6. US Department of Agriculture. Composition of foods: raw, processed, prepared, 1963-1991. Agricultural handbook no. 8-128-21 series. Washington DC: US Government Printing Office, 1992.

7. Clifford MN. Chlorogenic acids and other cinnamates - nature, occurrence and dietary burden. J Sci Food Agric 1999; 79:362-372.

8. Milder IE, Arts IC, van de Putte B, Venema DP, Hollman PC. Lignan contents of Dutch plant foods: a database including lariciresinol, pinoresinol, secoisolariciresinol and matairesinol. Br J Nutr 2005; 93:393-402.

9. Minamisawa M, Yoshida S, Takai N. Determination of biologically active substances in roasted coffees using a diode-array HPLC system. Anal Sci 2004; 20:325-8.

10. Greer F, Hudson R, Ross R, Graham T. Caffeine ingestion decreases glucose disposal during a hyperinsulinemic-euglycemic clamp in sedentary humans. Diabetes 2001; 50:2349-54.

11. Keijzers GB, De Galan BE, Tack CJ, Smits P. Caffeine can decrease insulin sensitivity in humans. Diabetes Care 2002; 25:364-9.

12. Yoshioka K, Kogure A, Yoshida T, Yoshikawa T. Coffee consumption and risk of type 2 diabetes mellitus. Lancet 2002; 360:703.

13. Acheson KJ, Zahorska-Markiewicz B, Pittet P, Anantharaman K, Jequier E. Caffeine and coffee: their influence on metabolic rate and substrate utilization in normal weight and obese individuals. Am J Clin Nutr 1980; 33:989-97.

14. Astrup A, Toubro S, Cannon S, Hein P, Breum L, Madsen J. Caffeine: a double-blind, placebo-controlled study of its thermogenic, metabolic, and cardiovascular effects in healthy volunteers. Am J Clin Nutr 1990; 51:759-67.

15. Bracco D, Ferrarra JM, Arnaud MJ, Jequier E, Schutz Y. Effects of caffeine on energy metabolism, heart rate, and methylxanthine metabolism in lean and obese women. Am J Physiol 1995; 269:E671-8.

16. Racotta IS, Leblanc J, Richard D. The effect of caffeine on food intake in rats: involvement of corticotropin-releasing factor and the sympatho-adrenal system. Pharmacol Biochem Behav 1994; 48:887-92.

17. Arion WJ, Canfield WK, Ramos FC, et al. Chlorogenic acid and hydroxynitrobenzaldehyde: new inhibitors of hepatic glucose 6-phosphatase. Arch Biochem Biophys 1997; 339:315-22.

18. Rodriguez de Sotillo DV, Hadley M. Chlorogenic acid modifies plasma and liver concentrations of: cholesterol, triacylglycerol, and minerals in (fa/fa) Zucker rats. J Nutr Biochem 2002; 13:717-726.

19. McCarty MF. A chlorogenic acid-induced increase in GLP-1 production may mediate the impact of heavy coffee consumption on diabetes risk. Med Hypotheses 2005; 64:848-53.

20. Kao WH, Folsom AR, Nieto FJ, Mo JP, Watson RL, Brancati FL. Serum and dietary magnesium and the risk for type 2 diabetes mellitus: the Atherosclerosis Risk in Communities Study. Arch Intern Med 1999; 159:2151-9.

21. Bhathena SJ, Velasquez MT. Beneficial role of dietary phytoestrogens in obesity and diabetes. Am J Clin Nutr 2002; 76:1191-201.

22. Graham TE, Hibbert E, Sathasivam P. Metabolic and exercise endurance effects of coffee and caffeine ingestion. J Appl Physiol 1998; 85:883-9.

23. Robinson LE, Savani S, Battram DS, McLaren DH, Sathasivam P, Graham TE. Caffeine ingestion before an oral glucose tolerance test impairs blood glucose management in men with type 2 diabetes. J Nutr 2004; 134:2528-33.

24. Salazar-Martinez E, Willett WC, Ascherio A, et al. Coffee consumption and risk for type 2 diabetes mellitus. Ann Intern Med 2004; 140:1-8.

25. Wu T, Willett WC, Hankinson SE, Giovannucci E. Caffeinated coffee, decaffeinated coffee, and caffeine in relation to plasma C-Peptide levels, a marker of insulin secretion, in u.s. Women. Diabetes Care 2005; 28:1390-6.

26. Comer SD, Haney M, Foltin RW, Fischman MW. Effects of caffeine withdrawal on humans living in a residential laboratory. Exp Clin Psychopharmacol 1997; 5:399-403.

27. Greenberg JA, Axen KV, Schnoll R, Boozer CN. Coffee, tea and diabetes: the role of weight loss and caffeine. Int J Obes Relat Metab Disord 2005.

28. Arnlov J, Vessby B, Riserus U. Coffee consumption and insulin sensitivity. JAMA 2004; 291:1199-201.

29. van Dam RM, Dekker JM, Nijpels G, Stehouwer CD, Bouter LM, Heine RJ. Coffee consumption and incidence of impaired fasting glucose, impaired glucose tolerance, and type 2 diabetes: the Hoorn Study. Diabetologia 2004; 47:2152-9.

30. Naismith DJ, Akinyanju PA, Szanto S, Yudkin J. The effect, in volunteers, of coffee and decaffeinated coffee on blood glucose, insulin, plasma lipids and some factors involved in blood clotting. Nutr Metab 1970; 12:144-51.

31. van Dam RM, Pasman WJ, Verhoef P. Effects of Coffee Consumption on Fasting Blood Glucose and Insulin Concentrations: Randomized controlled trials in healthy volunteers. Diabetes Care 2004; 27:2990-2.

32. Astrup A, Breum L, Toubro S, Hein P, Quaade F. The effect and safety of an ephedrine/caffeine compound compared to ephedrine, caffeine and placebo in obese subjects on an energy restricted diet. A double blind trial. Int J Obes Relat Metab Disord 1992; 16:269-77.

33. Westerterp-Plantenga MS, Lejeune MP, Kovacs EM. Body weight loss and weight maintenance in relation to habitual caffeine intake and green tea supplementation. Obes Res 2005; 13:1195-204.

34. Rakic V, Burke V, Beilin LJ. Effects of coffee on ambulatory blood pressure in older men and women: A randomized controlled trial. Hypertension 1999;33:869-73.

35. Altman DG, Schulz KF, Moher D, et al. The revised CONSORT statement for reporting randomized trials: explanation and elaboration. Ann Intern Med 2001; 134:663-94.

36. Segal KR, Van Loan M, Fitzgerald PI, Hodgdon JA, Van Itallie TB. Lean body mass estimation by bioelectrical impedance analysis: a four-site cross-validation study. Am J Clin Nutr 1988; 47:7-14.

37. Gavrila A, Chan JL, Yiannakouris N, et al. Serum adiponectin levels are inversely associated with overall and central fat distribution but are not directly regulated by acute fasting or leptin administration in humans: cross-sectional and interventional studies. J Clin Endocrinol Metab 2003; 88:4823-31.

38. McNamara JR, Schaefer EJ. Automated enzymatic standardized lipid analyses for plasma and lipoprotein fractions. Clin Chim Acta 1987;166:1-8.

39. Friedewald WT, Levy RI, Fredrickson DS. Estimation of the concentration of low-density lipoprotein cholesterol in plasma, without use of the preparative ultracentrifuge. Clin Chem 1972;18:499-502.

40. Stumvoll M, Mitrakou A, Pimenta W, et al. Use of the oral glucose tolerance test to assess insulin release and insulin sensitivity. Diabetes Care 2000;23:295-301.

41. Matthews DR, Hosker JP, Rudenski AS, Naylor BA, Treacher DF, Turner RC. Homeostasis model assessment: insulin resistance and beta-cell function from fasting plasma glucose and insulin concentrations in man. Diabetologia 1985; 28:412-9.

42. Matsuda M, DeFronzo RA: Insulin sensitivity indices obtained from oral glucose tolerance testing: comparison with the euglycemic insulin clamp. Diabetes Care 22:1462–1470, 1999

43. Swinburn BA, Boyce VL, Bergman RN, Howard BV, Bogardus C. Deterioration in carbohydrate metabolism and lipoprotein changes induced by modern, high fat diet in Pima Indians and Caucasians. J Clin Endocrinol Metab 1991; 73:156-65.

44. Juliano LM, Griffiths RR. A critical review of caffeine withdrawal: empirical validation of symptoms and signs, incidence, severity, and associated features. Psychopharmacology (Berl). 2004;176:1-29.

45. Verhoef P, Pasman WJ, Van Vliet T, Urgert R, Katan MB. Contribution of caffeine to the homocysteine-raising effect of coffee: a randomized controlled trial in humans. Am J Clin Nutr 2002;76:1244-8.

46. Noordzij M, Uiterwaal SPM, Arends LR, et al. Blood pressure response to chronic intake of coffee and caffeine: meta-analysis of randomized controlled trials. J Hyptertension 2005; 23:921-8.
